# Process Evaluation of a Sport-Based Voluntary Medical Male Circumcision Demand-Creation Intervention in Bulawayo, Zimbabwe

**DOI:** 10.1097/QAI.0000000000001172

**Published:** 2016-10-06

**Authors:** Jeff DeCelles, Rebecca B. Hershow, Zachary A. Kaufman, Katherine R. Gannett, Thandanani Kombandeya, Cynthia Chaibva, David A. Ross, Abigail Harrison

**Affiliations:** * Grassroot Soccer, Cape Town, South Africa;; † Department of Health Policy and Management, University of North Carolina, Chapel Hill, NC;; ‡ Faculty of Epidemiology and Population Health, London School of Hygiene and Tropical Medicine, London, United Kingdom;; § Faculty of Public Health and Policy, London School of Hygiene and Tropical Medicine, London, United Kingdom;; ‖ Grassroot Soccer Zimbabwe, Bulawayo, Zimbabwe;; ¶ National University of Science and Technology, Bulawayo, Zimbabwe; and; # Department of Behavioral and Social Sciences, Brown University School of Public Health, Providence, RI.

**Keywords:** male circumcision, demand creation, adolescents, HIV prevention

## Abstract

Grassroot Soccer (GRS) developed 2 brief and scalable voluntary medical male circumcision (VMMC) promotion interventions for males in Bulawayo, Zimbabwe, consisting of a 60-minute interactive, soccer-themed educational session with follow-up behavioral and logistical reinforcement. Both interventions were led by circumcised male community leaders (“coaches”) ages 18–30. “Make The Cut” (MTC) targeted adult males on soccer teams and “Make The Cut+” targeted boys in secondary schools. We conducted a process evaluation of MTC and Make The Cut+ to investigate perceptions of program impact, intervention components, and program delivery; participants' understandings of intervention content; and factors related to uptake.

**Methods:**

We conducted 17 interviews and 2 focus group discussions with coaches and 29 interviews with circumcised (n = 13) and uncircumcised participants (n = 16).

**Results:**

Findings demonstrate high program acceptability, highlighting the coach–participant relationship as a key factor associated with uptake. Specifically, participants valued the coaches' openness to discuss their personal experiences with VMMC and the accompaniment by their coaches to the VMMC clinic.

**Conclusions:**

Should the coach quality remain consistent at scale, MTC offers an effective approach toward generating VMMC demand among males.

## INTRODUCTION

Three randomized controlled trials (RCTs) have shown that voluntary medical male circumcision (VMMC) can reduce the risk of female-to-male transmission of HIV by 50%–60%.^1–3^ UNAIDS and WHO have stressed the importance and urgency of increasing uptake of VMMC among adolescent males, identifying schools and sports as 2 possible vehicles for intervention.^4^

Interest is growing internationally, particularly in Sub-Saharan Africa, in the use of sport for HIV prevention.^5^ Grassroot Soccer (GRS) is an international nongovernmental organization that uses the power of soccer in the fight against HIV and AIDS. Since 2002, GRS has delivered activity-based youth HIV prevention programs in Zimbabwe, which are now well established within government ministries, football clubs, and schools. This high level of community engagement provides GRS with the platform to combine the nation's passion for soccer with the fight against HIV and AIDS—particularly in encouraging VMMC uptake. Few studies have examined the effects of sport-based educational programming on VMMC uptake.^6^ There is an urgent need to scale up effective VMMC interventions in Zimbabwe, which is falling short of its target of 80% VMMC coverage by 2015 (approximately 203,419 VMMCs had been completed of the goal of 1.9 million people).^4,7^

Between 2012 and 2013, GRS developed 2 VMMC-promotion interventions, consisting of a 60-minute interactive, soccer-themed educational session with follow-up behavioral and logistical reinforcement (Table 1). One of these interventions, called “Make the Cut” (MTC), targeted men ages 18–30 from Bulawayo soccer teams. The second intervention, called “Make the Cut+” (MTC+), targeted secondary school boys ages 14–19 in Bulawayo. Two cluster RCTs were conducted to evaluate whether the interventions increased VMMC uptake among intervention participants. The Male Circumcision Uptake Through Soccer (MCUTS) RCT evaluating MTC found weak statistical evidence of an effect on VMMC uptake with 4.8% uptake among uncircumcised intervention participants, compared with 0.5% among control participants [odds ratio (OR) = 9.81, 95% CI: 0.93 to 103.2, *P* = 0.06].^8^ The MCUTS II RCT evaluating MTC+ found strong evidence of an effect on VMMC uptake with 12.2% uptake among uncircumcised intervention participants, compared with 4.6% among control participants (OR = 2.65, 95% CI: 1.19 to 5.86, *P* = 0.02).^9^

**TABLE 1.**
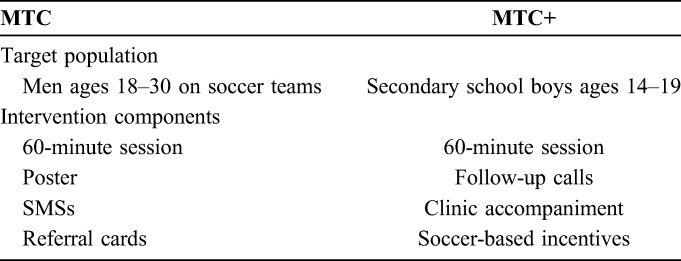
Summary of MTC Intervention Components

We present findings from a process evaluation of the MTC and MTC+ interventions. Process evaluations can help interpret outcome evaluations for social interventions by explaining the mechanisms and describing the context in which the intervention is delivered.^10,11^ We begin by discussing common themes from both MTC and MTC+ qualitative findings. We then explore differences in qualitative findings to generate a deeper understanding of the RCT results. Our objectives were to investigate perceptions of program impact, intervention components, and program delivery; participants' understandings of intervention content; and factors related to uptake. Our findings will contribute to evidence on VMMC demand-creation interventions for boys and men, informing future programming and scale-up.

## METHODS

### Intervention Design

Both MTC and MTC+ consisted of a 60-minute educational sessions comprised 3 activities: “Cut and Cover,” a soccer-based activity with known effectiveness in increasing knowledge of the health benefits of VMMC^12^; “Coach's Story,” a motivating personal story from the facilitator about his experience undergoing and recovering from VMMC; and a question-and-answer period. In line with social learning theory,^13^ the 60-minute session was delivered by circumcised GRS “coaches,” ages 18–30; use of role models has been identified as a key facilitator of VMMC uptake.^14^ Table 2 shows the intervention components of both MTC and MTC+.

**TABLE 2.**
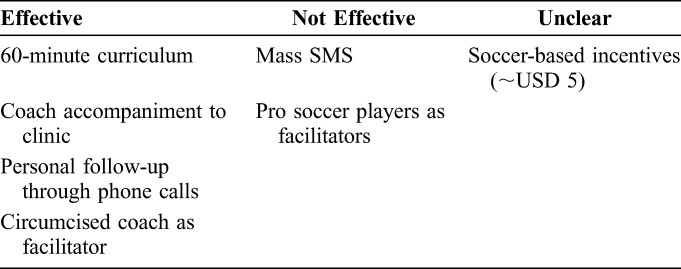
Overall Qualitative Lessons on Effective VMMC Uptake Intervention Components

A mix of professional soccer players and community members delivered the intervention to men belonging to professional and social soccer teams throughout Bulawayo. Over 4 months after MTC, participants received 23 SMSs that reinforced key messages, promoted adherence to post-operative healing instructions, and encouraged safe sexual behaviors. Before implementation, coaches took part in a 1-day training of coaches (TOC) workshop facilitated by GRS and Population Services International (PSI).

The revised version of the intervention developed in 2013, MTC+, consisted of the same 60-minute educational session but incorporated different forms of behavioral and logistical reinforcement: (1) phone-based follow-up by coaches to arrange transport to the VMMC clinic; (2) coach accompaniment to the VMMC clinic; and (3) soccer-based incentives valued at USD 5, which were offered to participants on VMMC completion. VMMC completion was determined by participants showing GRS coaches a stamped GRS referral card. VMMC clinic staff stamped the card if the participant completed the VMMC procedure at the clinic. GRS followed the guidelines in the PEPFAR Technical Considerations to avoid unethical procedures or appearance of coercion].^13^ MTC+ was delivered in Bulawayo secondary schools to boys ages 14–19 by circumcised GRS coaches. A similar TOC was conducted, with an additional day for a storytelling workshop.

The intervention was modified in April 2014 as PSI requested that all incentives be removed from the intervention for concerns that the incentives were unsustainable. Incentives were only offered to participants in 5 of the 13 intervention schools.

### Consent and Assent

Research assistants distributed consent forms to all participants; those who were under the age of 18 were required to obtain a signature of a parent or guardian to participate in the study. In addition, written assent was obtained from all participants.

Participants under the age of 16 who chose to get circumcised were required to bring a signed VMMC parental consent form to the clinic.

### Data Collection

Qualitative methods were used during the RCTs (Table 3). In total, 46 in-depth interviews (IDIs) and 2 focus group discussions (FGDs) were conducted. IDIs elicited individual experiences, opinions, and feelings.^15^ IDIs and FGDs specifically covered perceptions and acceptability of the intervention, perceptions of VMMC, influential factors in deciding whether to go for VMMC, and suggestions for program improvement.

**TABLE 3.**
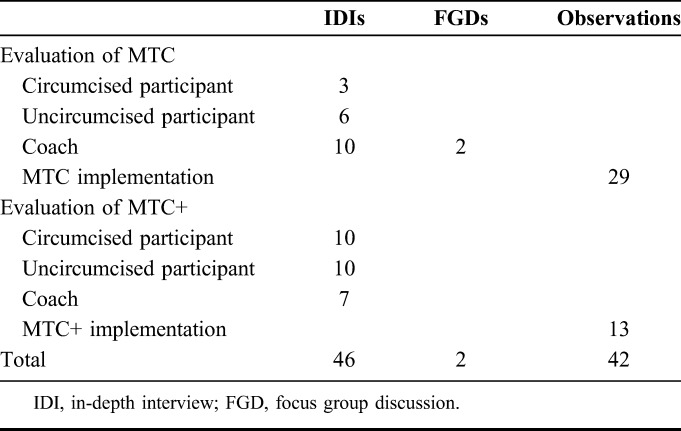
Qualitative Data Collected on MTC and MTC+

After the completion of the RCT of MTC, IDIs were conducted with participants (n = 9) and coaches (n = 10). MTC intervention-group participants who underwent VMMC within 45 days post-intervention (determined through clinical registers) were randomly selected for IDIs. Where possible, these participants were matched with a same-age participant from the same soccer team who did not undergo VMMC to enable comparison between similar participants. All active MTC coaches were selected for IDIs and participated in 2 FGDs. Research assistants conducted observation of the TOC and MTC interventions (n = 29) to monitor fidelity of the curriculum.

After the completion of the RCT of MTC+, the same qualitative methods were used. We conducted IDIs with 10 intervention-group participants who underwent VMMC within 45 days. These participants were purposively selected and matched with 10 same-age participants from the same school who did not undergo VMMC. Five of the 10 circumcised intervention youth participants we interviewed had received the soccer-based incentives. Research assistants conducted observation of the TOC workshop and MTC+ interventions (n = 13) in the same way.

IDIs and FGDs were conducted in English and Ndebele, depending on the participants' language proficiency. Research assistants fluent in both languages hosted the interviews and discussions. All IDIs and FGDs were audio recorded, transcribed verbatim, and translated into English when necessary.

### Data Analysis

Using NVivo10 software, a 4-person team coded the MTC transcripts, and a 5-person team coded the MTC+ transcripts. A preliminary coding scheme for MTC formative research was developed based on the topics in the interview and focus group guides, including experience in MTC, perceived impact of MTC, motivation, and barriers for VMMC, support, intervention components, materials, training, and suggestions for program improvement, including use of incentives. We used an applied thematic analysis approach to code and analyze the data, focused on thematic coding and identification of emergent themes.^16^

### Ethical Considerations

Ethical approval for the process evaluation was obtained from the Medical Research Council of Zimbabwe (MRCZ).

## RESULTS

### Emerging Themes for MTC and MTC+

#### Curriculum Components

Qualitative findings show that the curriculum offers a feasible approach toward VMMC promotion. Most MTC and MTC+ participants recalled both the Cut and Cover and Coach's Story activities in detail, citing increases in knowledge of dual benefits of VMMC and condom use.^18^ *“I learned that by circumcising you reduce the chances of contracting HIV, sexual transmitted disease, but it doesn't mean that you don't have to use protection”* (circumcised MTC+ participant).

Participants cited the Coach's Story as a motivational component of the curriculum. *“Because they explained to me what goes on and the fear disappeared and the stories about the pain and everything were not true. The way they told us about circumcision was encouraging and I thought I should try it”* (circumcised MTC participant).

Observation implementation showed that coaches sometimes skipped Cut and Cover with older participants (ages 30 and older), as the older participants did not want to participate in the activity, while MTC+ coaches consistently followed the steps in the curriculum.

#### Coach–Participant Relationship

Both MTC and MTC+ participants expressed appreciation for their coaches. MTC+ participants particularly valued their coaches' openness and honesty when discussing VMMC. *“To have someone who can actually tell you about [VMMC] was interesting, because all my life I have never had someone who can tell me about things, and for him to be open like that meant a lot”* (circumcised MTC+ participant). MTC+ participants also explained that they trusted their coaches and relied on their support.^17^ *“I created a special bond with [the coach] and I felt secure…because the guy made me do something that I never thought I could do”* (circumcised MTC+ participant). Coaches shared similar feelings about the importance of the relationships with participants, because if a participant decided to go for VMMC, he was *“[trusting] his life upon you”* (MTC+ coach).

### Unique Findings for MTC

#### Target Population

Although IDIs and FGDs demonstrated acceptability of curriculum components, participant age posed difficulty, both regarding intervention delivery and VMMC uptake. Coaches generally perceived older participants as uninterested in the soccer-based activity and less inclined to listen to key messages. *“They take you lightly”* (MTC coach). Moreover, older men were reported to lack motivation to undergo VMMC because they believed that HIV testing and VMMC would make little difference at their age. One coach described a common reaction of older participants: *“I have lived for 40 years and I'm not even HIV positive. So what am I protecting from?”* Numerous interviewees also suggested that participants—particularly older participants—were not interested in using PSI transport to go for VMMC as a group. One reason given by participants was that the status of a man who tests positive would be exposed when not proceeding directly into VMMC. Trial results reinforce these findings: MTC intervention participants ages 18–29 had higher VMMC uptake (5.1%) than intervention participants over 30 years (1.1%).^7^

#### Coach Type

A mix of professional soccer players and nonprofessional soccer players were trained as “coaches” to deliver MTC to explore whether status as a professional player is influential in increasing demand for VMMC. Qualitative findings suggested that experience and facilitation skill were more important characteristics of coaches than being a professional soccer player. No evidence of differences in uptake between the intervention group with professional-player facilitators and intervention group with nonprofessional-player facilitators was observed from MTC, although results trended toward lower uptake among teams with professional-player facilitators than in those with nonprofessional-player facilitators (OR = 0.55, 95% CI: 0.09 to 3.22).^8^

#### Follow-up SMSs

Follow-up SMSs received mixed reactions. Some participants enjoyed receiving SMSs, which they felt provided additional motivation to seek VMMC. Most could not remember the content of the SMSs, and some did not remember receiving any SMSs. Some coaches independently obtained phone numbers of interested participants and remained in contact after intervention delivery, which they felt was more influential in helping participants undergo VMMC than sending mass SMSs.

### Unique Findings for MTC+

#### Coach Follow-up

For some, the follow-up phone calls increased motivation to go for VMMC. *“I was 99.9%, but after [the follow-up] call, I was 100%”* (circumcised MTC+ participant). Coaches believed that phone calls were important in showing their commitment to participants. *“If you call the kids, they'll see you are also interested to see them go through with circumcision...if they see you are serious, they will also be serious about it”* (MTC+ coach).

Some coaches faced challenges reaching participants through phone (eg, if the phone number was incorrect), leading coaches to communicate with participants through WhatsApp (a free messaging platform for smartphones) or personally revisit participants' schools.^17^

#### Coach Accompaniment

Participants shared that they highly valued the coach accompaniment to the VMMC clinic and paid transport. *“[The coach] gave us a sense of security, a sense of safety, because if I was to go alone, maybe I would have turned around and came back home. But, with the coach, he's gone through it, he has the experience, and he knows more than you who have not done”* (circumcised MTC+ participant). Participants appreciated the opportunity during clinic trips to hear more about the VMMC procedure and talk informally, *“like friends, freely”* (circumcised MTC+ participant).^17^

#### Incentives

Circumcised participants were offered a choice between a t-shirt or a ticket to a local professional soccer match as further motivation to undergo VMMC. Because of a request from the VMMC service provider, incentive promotion and distribution was halted after implementation in 5 intervention schools, and incentives were not offered in the remaining 8 intervention schools. Although not statistically significant, uptake was slightly higher among participants who were offered the incentives (15.4%) than among those who were not (9.5%) (OR = 1.88, 95% CI: 0.68 to 5.23).^9^ Qualitative findings demonstrated mixed reactions to the incentives. Some participants felt that incentives increased their motivation to go for VMMC. *“I think [the incentives] just added some spice to something that was already nice…I know that I would have gone through the procedure even though there were no tickets”* (circumcised MTC+ participant). Others felt that the Coach's Story was a more important motivating factor.^17^ Overall acceptability was high for both the t-shirt and tickets as incentives. Some preferred the tickets because of their strong interest in soccer. Others preferred the t-shirt, which coaches believed stemmed from their desire to wear the same shirt as their coaches.

## DISCUSSION

Qualitative data demonstrate both MTC and MTC+ as acceptable and as offering an effective approach toward increasing VMMC uptake among males in Zimbabwe. Findings highlight the coach–participant relationship, coach accompaniment to the clinic, and age of participant as key factors in increasing participants' motivation to undergo VMMC. The data suggest that younger participants had higher appreciation and trust for their coaches. Many of the older MTC participants perceived circumcision as better suited for younger males, which seemed to prevent them from connecting with the coach and the messaging in MTC. Our findings align with recent research, which shows older males can be more difficult to engage through demand-creation initiatives.^14^ Further research is necessary to find a way to build on the successful components of MTC and MTC+ to develop a more effective VMMC demand-creation intervention for males beyond the adolescent years.

Components of the MTC+ intervention, including the educational session and coach accompaniment to the clinic, were valued by participants. In particular, Coach's Story seemed instrumental in generating discussion about VMMC and helping participants consider its pros and cons, because of the honest and intimate nature of the activity. VMMC demand-creation interventions should engage circumcised men as local role models and create a space where they can build a relationship with participants, share their personal experiences with VMMC, and accompany young men to the clinic. Incentives were viewed favorably, but because of the removal of incentives mid-trial, we were not able to assess whether they were instrumental to VMMC uptake. Although some research has shown the provision of food vouchers can increase VMMC uptake,^18^ further research is needed to determine the effectiveness of soccer-based incentives with boys.

Our study demonstrates the value of triangulating data from IDIs, FGDs, and clinical VMMC uptake data among men of varying ages. FGDs allowed us to obtain a range of opinions on the MTC intervention and understand community norms regarding VMMC.^19^ IDIs were valuable in allowing us to explore individual perceptions, feelings, and experiences.^17^ Both of these sources, when complemented by clinical VMMC uptake data, led to a thoughtful analysis of the MTC and MTC+ intervention components. Furthermore, the ability to compare qualitative data between MTC and MTC+ enabled us to understand how the changes to the intervention design (ie, target population, type of behavioral reinforcement) influenced participants' perceptions of intervention acceptability and motivation to undergo VMMC.

Among the limitations of this study is the fact that only 3 circumcised MTC participants were interviewed because of logistical challenges, limiting our ability to explore differences between circumcised and uncircumcised MTC participant responses. Findings could have been strengthened by conducting key informant interviews with officials from the ministries of education and health to explore acceptability and views on scale-up. Finally, as noted, the data are incomplete regarding the use of incentives.

GRS and implementing partners are looking to scale MTC+ throughout schools in Zimbabwe and other UNAIDS/WHO priority countries. Given the importance of the participant–coach relationship in a participant's decision to undergo VMMC, monitoring the quality of implementation during scale-up will be essential to ensure that this relationship remains strong.
